# Medical Heroism

**Published:** 1847-10

**Authors:** 


16. *Medical Heroism.*—There are few of our readers who do not remember the melancholy impression, made on the public mind by the disastrous expedition to the Niger when this was made known in England, through the newspapers. And none who remember this, can forget that pathetic passage in the story, which represented the noble conduct of the surgeon and the geologist of the expedition, when left alone in the far recesses of the Niger, amid their heroic companions, all stricken to death, or to death-like helplessness, by the fatal fever of the country. In this trying conjuncture, when the salvation of all on board depended upon the speedy removal of the ship from her actual position, Dr. M'William took the navigation on himself, steering with his own hands, and piloting the vessel through all the intricacies of the river, while his companion worked the engine below. There is something affecting, we had almost said, sublime, in the picture thus presented to the imagination, of these two solitary men of science, assuming offices so foreign to their past habits and knowledge, stripped of all exterior cognizance of their class, standing as humble workmen at the helm and furnace, toiling by day, watching by night, while the force of the stream and paddles was sweeping their ill-fated bark, freighted with their dying or dead companions through the manifold dangers of their unknown course. The author of the volume before us, (the Report on the Boa Vista Fever,) was the clear-headed and stout-hearted pilot who did this, the undoubted preserver of the ship and her surviving crew; and the slight and simple way in which he speaks of his own exertion, strikingiy illustrates the old truth, that "the brave man is ever modest."—*Brit. and For. Med. Review.*

What an entertaining book might be written upon "Medical Heroism!"—never more strikingly displayed than by the medical man in the battle, in the temptest, and in the pestilence, when neither reward nor glory is to be won, and philanthropy and duty alone prompt the exposure of a life, whose toils and sacrifices are to be so speedily forgotten. Such a work might do much towards obtaining a higher respect for the claims upon the public of our ill-requited profession.—*Annalist.*

